# Factors protecting Swiss nurses’ health during the COVID-19 pandemic: a longitudinal study

**DOI:** 10.1186/s12912-023-01468-6

**Published:** 2023-09-07

**Authors:** Jonathan Jubin, Philippe Delmas, Ingrid Gilles, Annie Oulevey Bachmann, Claudia Ortoleva Bucher

**Affiliations:** 1 La Source School of Nursing, HES-SO University of Applied Sciences and Arts Western Switzerland, Av. Vinet 30, Lausanne, 1004 Switzerland; 2 Lausanne University Hospital, Rue de Bugnon 21, Lausanne, CH-1011 Switzerland

**Keywords:** Nurses, Quality of life, Protective factors, Perceived stress, Social support, Resilience, COVID-19

## Abstract

**Background:**

The COVID-19 pandemic reached Europe in early 2020 and impacted nurses over a prolonged period, notably causing heavy work overloads. Exposure to sources of stress in such situations is inevitable, which can put nurses’ health at risk. The present study took a salutogenic approach to investigating nurses’ health and the principal factors protecting it found in the literature (i.e., resilience, post-traumatic growth, social support, and certain organizational factors), as well as how those elements evolved from February 2021 to September 2022.

**Methods:**

All nurses working at eight French-speaking Swiss hospitals who accepted to disseminate the study to their employees were invited to complete an online questionnaire at four time points (February 2021, September 2021, March 2022, and September 2022: T0, T1, T2, and T3, respectively) and respond to items measuring their health, factors protecting their health, and their perceived stress levels. Data were analyzed using random-intercept linear regression models.

**Results:**

A cumulated total of 1013 responses were collected over all measurement points (625 responses at T0; 153 at T1; 146 at T2; 89 at T3). Results revealed that nurses’ health had not changed significantly between measurements. However, their perceived stress levels, feelings of being supported by their management hierarchies, and belief that they had the means to deliver a high quality of work all diminished. At every measurement point, nurses’ health was negatively associated with perceived stress and positively associated with resilience, perceived social support, and the belief that they were provided with the means to deliver a high quality of work.

**Conclusion:**

Despite the difficult conditions caused by the pandemic, the factors recognized as protective of nurses’ health played their role. The lack of improvements in nurses’ health in periods when the pandemic’s effects lessened suggests that the pressure they were experiencing did not drop during these moments. This phenomenon may have been due to the need to clear backlogs in scheduled surgery and the work overloads caused by prolonged staff absences and nurses quitting the profession. Monitoring changes in nurses’ health is thus crucial, as is establishing measures that promote factors protective of their health. Organizational factors influencing nurses’ working conditions are also key and should not be neglected.

## Background

The COVID-19 pandemic let loose a sudden worldwide flood of patients requiring high levels of care and eventually led to high numbers of excess deaths [1]. As of 2019, numerous waves of the pandemic succeeded one another, sometimes accompanied by drastic preventive health measures, such as confinements, quarantines, and the closure of places of entertainment and recreation [2]. These successive waves generated enormous disruptions to nurses’ work, including the extra burden of huge numbers of patients, which sometimes went beyond wards’ capacities to provide them with the optimal conditions for quality care [3, 4]. Nurses in these situations faced such prolonged high levels of stress that it could have presented a risk to their health [5]. Indeed, many nurses found themselves incapable of working on and had to take sick leave, increasing the burdens on the staff remaining [6]. Moreover, in the troughs between the waves of the pandemic, many units had to make up their backlogs of non-urgent procedures, such as postponed scheduled surgery and follow-up with chronically ill patients. As such, nurses had no opportunity to recuperate from the build-up of pandemic-induced fatigue and stress [7]. These extremely demanding situations could exacerbate existing shortages of nursing staff and pressures on healthcare systems if they cause yet more highly qualified nurses to leave the profession [8].

Numerous studies have examined factors that can negatively affect nurses’ health in intense crisis situations, such as the SARS and MERS epidemics [9–11]. Literature reviews have highlighted many different factors, including psychological stress and heavy workloads, and they have presented diverse recommendations aiming to support nurses’ health and help them avoid too much exposure to stressful events [5, 12, 13]. However, in situations where exposure to sources of stress cannot be avoided, using a salutogenic approach can be an appropriate means of developing interventions that aim to reinforce nursing professionals’ individual resources and the resources available in their work settings [14, 15].

Salutogenic approaches concentrate on the factors favoring or protecting individuals’ health and well-being rather than on the factors that pose a risk to them [14, 15]. A salutogenic approach prioritizes using quality of life (QoL) as the principal indicator of health because it provides a holistic perspective on health and well-being that is not limited to whether a person is ill or not [15]. Based on the principles of salutogenesis, the present study chose nurses’ self-perceived QoL as its dependent variable, drawing insights from Neuman’s Systems Model, which recognizes the interconnectedness of stressors, adaptation, and holistic well-being [16, 17]. The World Health Organization defines QoL as “an individual’s perception of their position in life in the context of the culture and value systems in which they live and in relation to their goals, expectations, standards, and concerns” [18]. Furthermore, QoL is a concept fundamentally associated with professional commitment, a factor that has a significant influence on nurses choosing to remain in their chosen profession [19, 20]. QoL is recognized as being a multidimensional concept that cannot be simplified to individuals’ perceptions of their well-being or satisfaction with their life [21]. It is composed of four distinct dimensions. The physical dimension measures individuals’ levels of energy, but also of discomfort; the psychological dimension measures individuals’ self-image and self-body-image, as well as their positive and negative feelings about them; the social dimension measures the quality of individuals’ personal relationships and the social support they receive; and the environmental dimension measures diverse elements linked to individuals’ life contexts and settings, such as their physical safety, financial security, a healthy environment, and access to healthcare and transport [22]. In order to be able to design and introduce measures aimed at maintaining nursing staff’s health, it is crucial to identify the different elements supporting the different dimensions of their QoL and understand how those elements might change over time, particularly in the face of major crises like the COVID-19 pandemic.

Thus, the present study examines the principal variables described in the literature as playing important roles within strategies linked to protecting nurses from stress—variables that may have helped to reinforce their QoL during the pandemic [12, 23, 24]. These variables are (i) resilience, (ii) social support, and (iii) post-traumatic growth. Resilience describes an individual’s capacity to cope with stressful situations and bounce back after a traumatic event [25–27]. It plays a significant role in supporting health when individuals are confronted with sources of stress, not least in the face of a global health crisis [12, 28]. Social support refers to the psychological and material support provided to an individual by their entourage and which might help them cope with the stress they are experiencing by providing them with practical assistance, resources, and information [29–31]. The present study also examined workplace support from nurses’ management hierarchies and work colleagues [32–35]. Looking at post-traumatic growth allows us to measure the positive psychological changes that can occur after a traumatic event, such as greater confidence in oneself and one’s abilities and the development of new relationships with others [36, 37]. Post-traumatic growth is also notably associated with good mental health [38]. In addition to these variables, the present study examined nurses’ perception of their ability to provide quality care, something which the literature suggests can reduce the negative effects of the sources of stress that they must attempt to cope with [32].

Most of the studies on this subject have been transversal and, therefore, unable to measure variations in QoL throughout the pandemic or provide any evidence on the progressive or cumulative effects of the elements affecting QoL cited above. The present study, therefore, chose a longitudinal approach [39], and its goals were: (a) to measure temporal changes in nurses’ QoL scores, perceived stress levels, and factors protecting their health (resilience, social support, post-traumatic growth, and organizational elements); and (b) to evaluate how these protective factors and stress levels were associated with nurses’ QoL during the pandemic.

## Methods

### Design and population

To detect any changes in nurses’ quality of life that may have occurred over time, the present study used a longitudinal design with four measurement points: February to April 2021 (T0, during the pandemic’s third wave in Switzerland), September to November 2021 (T1, between its fourth and fifth waves), March to April 2022 (T2, at the end of the health restrictions), and September and October 2022 (T3). At T0, nurses from eight hospitals in Switzerland’s French-speaking regions^1^ received an e-mail from their management inviting them to complete an online questionnaire on their experiences of the COVID-19 pandemic. The e-mail contained an information sheet and a consent form that nurses had to read before participating in the study. Would-be participants gave their informed consent as their participation was voluntary, and they then self-administered the questionnaires. All nurses who worked at least half-time and understood French were eligible to participate. No exclusions based on departments were applied as even nurses who did not work directly with COVID-19 patients could have been affected by the consequences of the pandemic such as the lack of personnel or the fear of being contaminated. Their institutions allowed them to complete their questionnaires during their working hours. Nurse managers, nurses who had had no professional activities in their institution during the pandemic or had been nursing students during that time, were not eligible to take part in the study. More details are provided in the study protocol [40].

From an available population of 2710 nurses working in the eight hospitals taking part in the study at T0, we collected questionnaire responses from 627 nurses (response rate: 23.1%). Among these, 345 agreed to be contacted again at subsequent data collection points. We contacted these 345 nurses again at T1, T2, and T3 using an e-mail containing a link to the same online questionnaire. At T1, 153 nurses completed the questionnaire (response rate: 44.3%); at T2, 176 nurses completed it (response rate: 51.0%); and at T3, 103 nurses completed it (response rate: 29.9%).

Data were coded so that only the researchers in charge of contacting participants at each data collection point had access to participants’ e-mail addresses. Participants’ data from the different collection time points were associated using unique random alphanumerical identifiers generated by the survey software. The questionnaire took between 25 and 35 min to complete.

## Measures

All Cronbach’s α [41] and descriptive statistics related to the following scales are reported in Table 1.

**Table 1:** Descriptive statistics for numerical variables at each data collection point

Variable (response range)	Mean (SD) T0 (N = 625)	Mean (SD) T1 (N = 153)	Mean (SD) T2 (N = 146)	Mean (SD) T3 (N = 89)	Cronbach’s α range across measurement points	Effect of measurement point
Physical QoL (0–100)	69.17 (16.29)	67.18 (18.86)	67.93 (17.54)	70.45 (18.10)	0.78–0.84	T0 vs. T1: β = -0.10, *p* = .100 T0 vs. T2: β = -0.04, *p* = .479 T0 vs. T3: β = -0.05, *p* = .543
Psychological QoL (0–100)	65.20 (16.31)	64.85 (18.07)	65.33 (16.68)	66.23 (18.48)	0.77–0.82	T0 vs. T1: β = -0.04, *p* = .532 T0 vs. T2: β = 0.04, *p* = .516 T0 vs. T3: β = 0.01, *p* = .921
Social QoL (0–100)	67.40 (21.29)	66.12 (21.32)	65.30 (21.95)	64.98 (21.95)	0.69–0.78	T0 vs. T1: β = -0.07, *p* = .234 T0 vs. T2: β = -0.10, *p* = .115 T0 vs. T3: β = -0.13, *p* = .098
Environmental QoL (0–100)	70.58 (14.11)	71.50 (16.02)	72.46 (14.97)	72.61 (15.81)	0.75–0.84	T0 vs. T1: β = -0.04, *p* = .324 T0 vs. T2: β = 0.08, *p* = .187 T0 vs. T3: β = 0.02, *p* = .752
Perceived stress (1–5)	2.84 (0.61)	2.81 (0.68)	2.76 (0.68)	2.63 (0.68)	0.90–0.93	T0 vs. T1: β = 0.03, *p* = .592 **T0 vs. T2: β = -0.13**, ***p*** **= .034** **T0 vs. T3: β = -0.19**, ***p*** **= .015**
Post-traumatic growth (1–6)	3.25 (1.02)	3.32 (1.00)	3.37 (1.04)	3.30 (0.96)	0.87–0.88	T0 vs. T1: β = 0.02, *p *= .743 T0 vs. T2: β = 0.08, *p *= .280 T0 vs. T3: β = 0.02, *p *= .805
Resilience (1–5)	3.67 (0.63)	3.80 (061)	3.73 (0.61)	3.74 (0.69)	0.88–0.90	T0 vs. T1: β = 0.05, *p *= .308 T0 vs. T2: β = 0.05, *p *= .393 T0 vs. T3: β = 0.02, *p *= .808
Perceived social support (1–7)	5.76 (1.08)	5.67 (1.02)	5.65 (1.05)	5.58 (1.11)	0.92–0.94	T0 vs. T1: β = -0.09, *p* = .125 T0 vs. T2: β = -0.07, *p* = .219 **T0 vs. T3: β = -0.22**, ***p*** **= .002**
Support from management hierarchy (1–6)	4.41 (1.11)	4.25 (1.28)	4.19 (1.26)	4.33 (1.20)	0.86–0.92	**T0 vs. T1: β = -0.18**, ***p*** **= .008** **T0 vs. T2: β = -0.22**, ***p*** **= .004** T0 vs. T3: β = -0.11, *p* = .218
Support from colleagues (1–6)	4.70 (0.83)	4.68 (0.80)	4.57 (0.93)	4.58 (0.85)	0.80–0.84	T0 vs. T1: β = -0.04, *p* = .557 T0 vs. T2: β = -0.12, *p* = .103 T0 vs. T3: β = -0.14, *p* = .112
Quality of work (1–5)	4.61 (0.74)	4.41 (0.97)	4.35 (0.91)	4.52 (1.02)	0.80–0.94	**T0 vs. T1: β = -0.22**, ***p*** **= .002** **T0 vs. T2: β = -0.32**, ***p*** **< .001** T0 vs. T3: β = -0.15, *p* = .087

β: standardized regression coefficient from random-intercept linear regression models with time measurement point as only independent variable. **Bold**: statistically significant results at *p* < .05.

### Outcome variables

The *World Health Organization Quality of Life–BREF (WHOQOL-BREF)* [18, 42] includes 26 items rated on five-point Likert scales, with high scores indicating high QoL. This scale measures four domains of QoL: physical (seven items), psychological (six items), social (three items), and environmental (eight items). Its validity and reliability were validated in French with Cronbach’s α ranging from 0.59 to 0.74 [42, 43]. In the present study, satisfying reliability was observed for all dimensions at each time points with Cronbach’s α ranging from 0.69 to 0.84.

### Determinants

The *Perceived Stress Scale (PSS)* [44, 45] measures the degree to which individuals perceive their life as unpredictable, uncontrollable, and painful [46]. It includes 14 items rated from 1 to 5, with high values indicating a high level of perceived stress. The French translation was validated and showed good reliability with Cronbach’s α = 0.84 [45, 47]. It was even higher in the present study as it ranged from 0.90 to 0.93.

The *Post-Traumatic Growth Inventory–Short Form (PTGI–SF)* [37, 48] measures positive psychological change following a traumatic event. It includes ten items rated from 1 to 6, with high values indicating having experienced a lot of change. The French version was validated and displayed good reliability with Cronbach’s α > 0.90 [48, 49] which was echoed in the present study with Cronbach’s α ranging from 0.87 to 0.88.

The *Connor–Davidson Resilience Scale (CD-RISC®)* measures how individuals are able to bounce back after a setback or a traumatic event [26, 50, 51]. It includes ten items rated from 1 to 5, with high values indicating a high level of resilience. The French translation had been validated and showed good reliability with Cronbach’s α = 0.86 [50, 52, 53]. Reliability was as good in the present study Cronbach’s α ranging from 0.88 to 0.90.

The *Multidimensional Scale of Perceived Social Support (MSPSS)* measures the psychological and material support that individuals perceive they receive from their family, friends, and significant other [31, 54]. It includes 12 items rated from 1 to 7, with high values indicating a high level of social support. The validity and reliability of the scale were validated in French with Cronbach’s α ranging from 0.91 to 0.94 [54]. In the present study, reliability was also good for all dimensions at each time points with Cronbach’s α ranging from 0.92 to 0.94.

The *Copenhagen Psychosocial Questionnaire (COPSOQ)* measures 24 dimensions assessing psychosocial risk in the workplace [32, 55, 56]. The present study incorporated three dimensions: social support from colleagues (three items rated from 1 to 6), social support from supervisors (three items rated from 1 to 6), and quality of work (i.e., the ability to deliver work of quality; two items rated from 1 to 5). In our analyses, high values indicate high support or quality of work [56]. The questionnaire was validated in several languages, including French and showed good psychometric properties [32, 57]. Good reliability was observed in the present study with Cronbach’s α ranging from 0.80 to 0.94.

### Sociodemographic and control variables

The questionnaire asked for participants’ gender (man, woman, and ‘I define myself otherwise’), age category (18–29, 30–39, 40–49, and < 50 years old), and how long they had been working in their current position (< 2, 2–5, and > 5 years). Participants were also asked whether they had been reassigned to any units other than their usual one during the pandemic (no, yes for < 1 month, yes for > 1 month, and yes, multiple times) and if they had been exposed to COVID-19 during their work (direct exposure: worked in a COVID-specific unit; indirect exposure: worked in a non-COVID-specific unit that admitted some COVID patients; and no exposure: no COVID patients were admitted to the unit).

### Data analysis

Returned questionnaires with more than 50% of responses missing were removed from all analyses (T0: 2 removed; T1: 0 removed; T2: 30 removed; T3: 14 removed), leaving 625 exploitable questionnaires at T0, 153 at T1, 146 at T2, and 89 at T3. All the variables were treated as continuous except for the sociodemographic variables, which were treated as dichotomized (two modalities) or binary variables (three modalities or more). First, descriptive statistics of all the variables were calculated for each measurement point and these were compared using Chi-square tests of independence for categorical variables and random-intercept linear regression models testing the association with measurement time for numerical variables. After checking the linearity and normality assumptions, we used hierarchical random-intercept linear regression models with the four domains of QoL as dependent variables. This enabled us to compute models even if some participants had not responded at each time point [58]. To enable model comparisons, we imputed the remaining missing values (2.82% of data) using the mean for numerical variables and the mode for categorical variables. All numerical variables were standardized to enable a comparison of coefficients. Predictors were added to the model block by block, starting with the effects of the measurement point (block 1), followed by sociodemographic variables (block 2), COVID-19-related variables (block 3), and, finally, perceived stress and protective factors (block 4). Models were compared based on their deviance. They were fitted using R 4.2.2 and the lme4 (v.1.1–31) package [59]. Multicollinearity among the dependent variables was checked using the variance inflation factor (VIF), but no problematic collinearity emerged (all VIFs < 3) [60]. Statistical significance was set at *p* < .050.

## Results

### Descriptive results

Table 2 shows descriptive statistics for categorical and numerical variables, respectively. Results showed that at each measurement point, most participants were women (83.1–87.0%), were aged from 30 to 39 (24.7–33.0%) or 40–49 (24.0–32.6%), and had worked in their position for more than five years (51.7–65.2%). A majority had been exposed to COVID-19 indirectly by working in a unit that admitted some COVID-19 patients but was not fully dedicated to treating them (45.0–50.6%), and many were exposed directly by working in COVID-19-specific units (34.9–39.7%). Lastly, more than one third of participants were reassigned to a different unit at least once during the pandemic (33.1–44.9%).

**Table 2:** Descriptive statistics for categorical variables at each data collection point

Variable and modality	% T0 (N = 625)	% T1 (N = 153)	% T2 (N = 146)	% T3 (N = 89)	Effect of measurement point
Gender: Woman	85.9	83.7	87.0	83.1	*χ*^2^(3) = 1.15, *p* = .764
Gender: Man	13.6	15.0	12.3	16.9	
Age: 18–29	20.0	13.7	10.3	9.0	*χ*^2^(9) = 23.15, *p* = .006
Age: 30–39	33.0	30.1	31.5	24.7	
Age: 40–49	24.0	31.4	32.2	32.6	
Age: ≥ 50	22.7	24.8	26.0	33.7	
Time in current position: < 2 years	24.0	13.7	13.0	9.0	*χ*^2^(6) = 22.33, *p* = .001
Time in current position: 2–5 years	23.7	27.5	26.0	25.8	
Time in current position: > 5 years	51.7	58.8	61.0	65.2	
COVID-19 exposure: None	15.2	15.7	15.8	12.4	*χ*^2^(6) = 2.23, *p* = .897
COVID-19 exposure: Indirect	45.0	47.1	49.3	50.6	
COVID-19 exposure: Direct	39.7	37.3	34.9	37.1	
Reassigned: No	66.9	62.7	56.2	55.1	*χ*^2^(9) = 12.82, *p* = .171
Reassigned: Short	10.4	9.15	11.0	11.2	
Reassigned: Long	9.1	12.4	15.1	15.7	
Reassigned: Multiple	13.0	15.0	17.8	18.0	

Note: some percentages do not add up to 100 because of non-responses.

As each progressive data collection point, the proportion of older respondents increased (χ^2^(9) = 23.15, *p* = .006), as did the proportion of respondents who had worked in their current position for longer (χ^2^(6) = 22.33, *p* = .001). These changes are unsurprising, however, since age and time spent in one’s current position depend directly on advancing time. Perceived stress was lower at T2 and T3 than at T0 (β_T2_ = -0.13, *p* = .034; β_T3_ = -0.19, *p* = .015), and perceived social support was lower at T3 than at T0 (β_T3_ = -0.22, *p* = .002). Moreover, support from management hierarchies (β_T1_ = -0.18, *p* = .008; β_T2_ = -0.22, *p* = .004) and the perceived quality of the work performed (β_T1_ = -0.22, *p* = .002; β_T2_ = -0.32, *p* < .001) decreased at T1 and T2. Trends for other variables, including all the dimensions of QoL, were not significant.

### Results from hierarchical random-intercept linear regression models

Results from the hierarchical random-intercept linear regression models are presented in Table 3. We first describe results consistent across all the dimensions of QoL and then the results specific to each separate dimension of QoL.

Overall, it appeared that none of the dimensions of QoL was associated with block 1 (time point; χ^2^(3) < 4.50, *p*s > 0.050), block 2 (demographics; χ^2^(6) < 11.00, *p*s > 0.050), or block 3 (reassignment and COVID-19 exposure; χ^2^(5) < 8.00, *p*s > 0.050) as those blocks did not significantly improve the dimension models, with the exceptions of block 2 improving the social QoL model (χ^2^(6) = 16.30, *p* = .012) and block 3 improving the psychological QoL model (χ^2^(5) = 15.22, *p* = .009).

Additionally, block 4 (perceived stress and QoL resources) significantly improved the models for all four QoL dimensions (χ^2^(7) range: 340.19–629.94, *p*s < 0.001), with low perceived stress (βs < -0.27, *p*s < 0.001) and high perceived social support (βs > 0.14, *p*s < 0.001) being consistently associated with high a level of QoL. The associations between other specific variables and the QoL dimensions varied across dimensions and are detailed in the next sections.

**Table 3:** Hierarchical random-intercept linear regression models

Block	Variables	QoL Physical		QoL Psychological		QoL Social		QoL Environment	
		β	se	β	se	β	se	β	se
Block 1	Measurement point: T1	-0.05	0.05	0.00	0.05	-0.04	0.06	0.03	0.06
	Measurement point: T2	-0.05	0.06	0.01	0.05	-0.11	0.06	0.11	0.06
	Measurement point: T3	-0.06	0.07	-0.03	0.06	-0.09	0.07	0.03	0.07
	Change in deviance	*χ*^2^(3) = 2.96 *p* = .398		*χ*^2^(3) = 1.13 *p* = .770		*χ*^2^(3) = 4.41 *p* = .220		*χ*^2^(3) = 3.23 *p* = .358	
Block 2	Gender: Man	0.09	0.08	0.04	0.08	-0.23**	0.09	-0.07	0.09
	Age: 30–39	-0.02	0.08	0.10	0.08	-0.14	0.09	-0.10	0.09
	Age: 40–49	-0.04	0.09	0.16	0.09	-0.19	0.10	0.05	0.10
	Age: ≥ 50	-0.21*	0.10	0.17	0.09	-0.33**	0.10	0.05	0.11
	Time in current position: 2–5 years	-0.03	0.07	0.01	0.06	0.08	0.07	0.11	0.08
	Time in current position: > 5 years	0.01	0.07	0.00	0.07	0.15	0.08	0.14	0.08
	Change in deviance	*χ*^2^(6) = 3.05 *p* = .802		*χ*^2^(6) = 10.80 *p* = .095		*χ*^2^(6) = 16.30 *p* = .012		*χ*^2^(6) = 9.53 *p* = .146	
Block 3	COVID-19 exposure: Indirect	0.01	0.07	0.09	0.06	0.06	0.07	-0.02	0.08
	COVID-19 exposure: Direct	0.04	0.08	0.13	0.07	0.07	0.08	0.14	0.08
	Reassignment: Short	-0.09	0.08	-0.01	0.07	0.10	0.08	0.06	0.08
	Reassignment: Long	-0.01	0.07	0.08	0.08	0.05	0.09	0.03	0.09
	Reassignment: Multiple	-0.06	0.07	-0.10	0.06	0.01	0.07	0.00	0.07
	Change in deviance	*χ*^2^(5) = 6.81 *p* = .235		*χ*^2^(5) = 15.22 *p* = .009		*χ*^2^(5) = 4.01 *p* = .548		*χ*^2^(5) = 7.94 *p* = .160	
Block 4	Perceived stress	-0.49***	0.03	-0.43***	0.03	-0.27***	0.03	-0.35***	0.03
	Post-traumatic growth	0.00	0.02	0.06*	0.02	0.04	0.03	-0.02	0.03
	Resilience	0.04	0.03	0.15***	0.03	0.07*	0.03	0.07*	0.03
	Perceived social support	0.14***	0.03	0.23***	0.03	0.41***	0.03	0.16***	0.03
	Support from management hierarchy	0.03	0.03	-0.02	0.02	-0.03	0.03	0.03	0.03
	Support from colleagues	0.03	0.03	0.04	0.02	0.06*	0.03	0.06*	0.03
	Quality of work	0.11***	0.03	0.10***	0.02	-0.01	0.03	0.13***	0.03
	Change in deviance	*χ*^2^(7) = 479.83 *p* < .001		*χ*^2^(7) = 629.94 *p* < .001		*χ*^2^(7) = 387.66 *p* < .001		*χ*^2^(7) = 340.19 *p* < .001	

QoL: Quality of life; β: standardized regression coefficient; se: standard error. *p*: 0 ‘***’ 0.001; ‘**’ 0.010; ‘*’ 0.050. All standardized regression coefficients are from the complete analyses that included all the blocks.

### Results specific to physical QoL

Only block 4 (perceived stress and QoL resources) had a significant impact on the physical QoL model’s deviance (χ^2^(7) = 479.83, *p* < .001). In addition to the associations mentioned above, the ability to produce work of high quality was also associated with physical QoL (β = 0.11, *p* < .001). In other words, when participants felt that they were provided with the adequate means to do their work properly, they tended to report a better physical QoL.

### Results specific to psychological QoL

Block 3 (reassignment and COVID-19 exposure) improved the psychological QoL model significantly (χ^2^(5) = 15.22, *p* = .009). Participants who had been reassigned multiple times during the pandemic reported a lower psychological QoL than those who had never been reassigned (β = -0.26, *p* = .001). However, these effects no longer held once block 4 was added to the model.

Block 4 also improved this model’s deviance (*χ*^2^(7) = 629.94, *p* < .001). In addition to the associations mentioned above, post-traumatic growth (β = 0.06, *p* = .013), resilience (β = 0.15, *p* < .001), and quality of work (β = 0.10, *p* < .001) were all associated with psychological QoL. Participants who reported high post-traumatic growth, high resilience, or felt they were provided with the adequate means to do their work properly, tended to report a better psychological QoL.

### Results specific to social QoL

Block 2 improved the model’s deviance (*χ*^2^(6) = 16.30, *p* = .012). Men tended to show a lower social QoL than women (β = -0.25, *p* = .025), and all the age categories reported lower social QoL than the 18–29 group (βs < -0.25, ps < 0.050). This effect remained significant for men and those aged ≥ 50, even in the complete model including all four blocks.

Block 4 improved the model’s deviance (*χ*^2^(7) = 387.66, *p* < .001). In addition to the associations mentioned above, resilience (β = 0.07, *p* = .031) and support from colleagues (β = 0.06, *p* = .035) were associated with social QoL. Participants who reported high resilience or high support from colleagues tended to report a better social QoL.

### Results specific to environmental QoL

Only block 4 had a significant impact on the environmental QoL model’s deviance (*χ*^2^(7) = 340.19, *p* < .001). In addition to the associations mentioned above, resilience (β = 0.07, *p* = .023), support from colleagues (β = 0.06, p = .028), and quality of work (β = 0.13, *p* < .001) were associated with environmental QoL. When participants reported high resilience, high support from colleagues, or felt like they were provided with adequate means to do their work properly, they tended to report a greater environmental QoL.

## Discussion

The present study selected a salutogenic approach to investigate the changing health of French-speaking Switzerland’s nurses during the COVID-19 pandemic (February 2021 to October 2022) and measured different dimensions of their quality of life (QoL) [14, 15], recognized factors protective of health taken from the literature, and their perceived levels of stress. Overall, and independently of the time points at which measurements were made, findings showed that perceived levels of stress were negatively associated with all the dimensions of QoL. Social support and resilience were positively associated with three dimensions of QoL but not with physical QoL. Finally, being given the means to perform high-quality work was also associated with three dimensions but not with social QoL. Furthermore, exposure to COVID-19 in the workplace and being reassigned to work in a different clinical unit were not associated with QoL. The only exception to this was among nurses who were reassigned to other units numerous times, which was associated with a worse psychological QoL when perceived stress and other protective factors were not included in the model. Findings also revealed that none of the four dimensions of QoL changed significantly between measurement points. Perceived stress, however, diminished significantly, as did the feelings of being supported by one’s management hierarchy and being provided with the resources to perform quality work.

The impact of stress on nurses’ health has been investigated many times because this profession has been exposed to other sources of stress numerous times before the present pandemic [61–65]. The negative effects of stress on both physical and mental health are well-known, with notable impacts on gastrointestinal, immune system, and cardiovascular function, as well as on neurological and psychiatric health disorders [66]. Social support’s role as a protective factor against stress’s undesirable effects on mental health and as a factor in QoL is well documented [67, 68]. The support provided by one’s entourage, given in the form of aid and information, helps individuals cope with situations of uncertainty and perceived stress [69, 70]. Resilience has also long been known as a protective factor for health. Resilient individuals may have a tendency to use more active strategies more often, such as positive reinterpretation and acceptance [71–73]. During the pandemic, these types of strategies may have helped them to find more effective solutions for managing this unique situation. It is interesting to note that our findings suggest that the factors recognized as being protective of health before the pandemic continued to play that role during it. The global health crisis only consolidated a pre-existing situation of nursing stress and heavy professional burdens.

The association between the quality of the work performed and QoL echoes the literature revealing that nurses’ professional satisfaction is linked to the quality of the care that they provide [74]. Providing high-quality care may make nurses feel more professionally satisfied and develop their feelings of having truly accomplished something, creating a positive feedback loop [75, 76]. This is a particularly interesting finding for care institutions to consider. Indeed, providing nurses with a high-quality working environment and resources may support their QoL and improve the quality of the care that they are able to give. On the contrary, post-traumatic growth was not associated with QoL. This suggests that although some of our study participants reported positive changes due to the pandemic, these were not all replicated in their QoL. Because the study took place during the pandemic, it is also possible that any impact from all these changes will only occur later.

The longitudinal results showed that none of the four dimensions of QoL investigated significantly changed between measurement points. One might have expected some improvement as the epidemiological pressure eased from 2021 to 2022, notably due to the successful development of vaccines and the subsequent reduction in severe cases of COVID-19. However, this might be explained by some of the pandemic’s consequences. Indeed, existing shortages of nursing personnel were exacerbated by numerous staff leaving their jobs and the profession or going on long-term leave during this period. This left the remaining staff with even heavier burdens [77]. Moreover, the pandemic caused the postponement of numerous non-urgent surgical interventions that had to be rescheduled in the lulls between COVID-19’s successive waves. As such, nurses never got the time to recuperate [7]. The stability observed in nurses’ QoL is coherent with the findings indicating that their perceived stress levels diminished but that their capacity to perform their work to a high standard diminished too. Indeed, because perceived stress is negatively associated with QoL, lower perceived stress could have led to increased QoL. Similarly, a reduction in the quality of nurses’ work could have been accompanied by a lower QoL. These two phenomena could have compensated for each other and resulted in a stable QoL over time. Nevertheless, as noted by a reviewer, the lack of improvement of QoL over time might not have been caused solely by the COVID-19 pandemic. This phenomenon might also indicate that the stress under which nurses were already before the pandemic has become chronic, preventing them from recuperating.

The final point highlighted by the present findings is that nurses reported a reduction in support from their hierarchical superiors. Interrogated nurses suggested that the initial waves of COVID-19 had such a significant impact on care that hospital institutions made large amounts of resources available to provide nurses with the robust support they needed (manuscript in preparation). Yet, those resources gradually fell away again over time, despite the continued presence of the pandemic. The literature has shown that managers had to adapt extremely fast to cope with this almost completely unexpected and constantly evolving situation. Decisions had to be taken on the basis of incomplete information and evidence, and the information communicated about the pandemic was sometimes inconsistent [78]. One study in Switzerland revealed that frontline nurse managers had to reconcile the sometimes-contradictory demands made by their care teams and higher management echelons and that management roles and styles changed significantly during this crisis [79]. Faced with these numerous demands, it is possible that frontline nurse managers tired themselves out or that institutional rules and recommendations progressively reduced their ability to support frontline nurses.

Based on the findings of the present study, several recommendations for future research studies in the field of nurses’ health and well-being emerge. Firstly, further investigation could delve into the specific mechanisms that contribute to the observed associations between nurses’ health, resilience, and perceived social support. Secondly, longitudinal studies with extended follow-up periods could shed light on the long-term effects of the COVID-19 pandemic on nurses’ health and their evolving perceptions of support and work-related capabilities. Lastly, comparative studies across diverse healthcare settings and cultural contexts would provide a comprehensive understanding of how the identified protective factors interact within varying nursing environments, contributing to a more nuanced and globally applicable framework for nurses’ well-being.

### Study limitations

This study’s principal limitation was attrition. The 75% attrition rate between T0 and T1 was far above the 20% envisioned in the study protocol [40]. Despite recontacting at T2 and T3 all the participants who had agreed to be recontacted at T0, the sample diminished significantly between measurement points. It is possible that the heavy workloads imposed on nurses by the pandemic made responding to a survey feel like an additional burden. Furthermore, it is possible that those participants who continued to respond at each measurement point were those in the best health, as described by the healthy worker effect [80, 81]. Thus, our findings should be interpreted with care because they may have overestimated the nurses’ true health.

Another limitation was that the Perceived Stress Scale measures the overall level of stress that an individual might perceive, not just the stress induced by their work. Nevertheless, because the COVID-19 pandemic affected every aspect of nurses’ lives, it is important to consider the perceived stress in their private lives too, in order to fully evaluate their health.

Finally, the study mainly focused on social support and some of the internal factors protecting nurses’ health. It is important to note that other factors can affect nurses’ health, such as work organization and management practices [82].

## Conclusions

Nurses are exposed to many health-threatening sources of stress in the course of their work, and the COVID-19 pandemic only made the situation worse. In such a context, understanding which factors might protect nurses’ health is crucial. Our results showed that perceived stress was the primary determinant of the four dimensions of quality of life (QoL) that we examined, but social support, resilience, and the ability to perform work of high quality were also consistently associated with most of these dimensions. Moreover, none of the dimensions of QoL evolved significantly across the study’s four measurement points, which suggests that despite improvements to the overall health situation in successive waves of the pandemic, its repercussions might have continued to affect nurses’ QoL. Thus, it is important for managers to monitor nurses’ real, everyday working conditions and base their decisions on these rather than on reactions to emergency decision-making at any one specific time. Additionally, measures that help nurses to benefit from social support outside of their work settings, such as promoting an appropriate work–life balance, and measures that foster resilience, should all be encouraged. Finally, nurses should be given the means to perform high-quality work, which is likely to result in more professionally satisfied nurses and high-quality care. Only by sustaining a healthy and resilient workforce can healthcare systems cope with crises such as the COVID-19 pandemic.

## Data Availability

The datasets generated and analysed during the current study are not publicly available due to the presence of health information but are available from the corresponding author on reasonable request.

## Footnotes

1 Participating institutions included 3 public hospitals, 4 private clinics, and 1 university hospital, of which 7 were located in urban areas and 1 in a rural area. Nursing staff was 400 or more in 3 institutions and less than 400 in the other 5.

## Abbreviations

QoL: Quality of life
